# Protective role of engineered extracellular vesicles loaded quercetin nanoparticles as anti-viral therapy against SARS-CoV-2 infection: A prospective review

**DOI:** 10.3389/fimmu.2022.1040027

**Published:** 2022-12-08

**Authors:** Alok Raghav, Richa Giri, Saurabh Agarwal, Sanjay Kala, Goo-Bo- Jeong

**Affiliations:** ^1^ Department of Anatomy and Cell Biology, College of Medicine, Gachon University, Incheon, South Korea; ^2^ Multidisciplinary Research Unit, GSVM Medical College, Kanpur, Uttar Pradesh, India; ^3^ Kailashpat Singhania (KPS), Institute of Medicine, GSVM Medical College, Kanpur, Uttar Pradesh, India; ^4^ Department of Surgery, GSVM Medical College, Kanpur, Uttar Pradesh, India; ^5^ Department of Anatomy and Cell Biology, College of Medicine, Gachon University, Incheon, South Korea

**Keywords:** Quercetin, extracellular vesicle, SARS CoV2, nanoparticle, therapeutic target

## Abstract

Quercetin (QCT) is a naturally occurring phenolic flavonoid compound with inbuilt characteristics of antioxidant, anti-inflammatory, and immune protection. Several recent studies have shown that QCT and QCTits nanoparticles have therapeutic potential against severe acute respiratory syndrome coronavirus-2 (SARS-CoV-2) infection. Novel therapeutics also include the implication of extracellular vesicles (EVs) to protect from SARS-CoV-2 viral infection. This article highlighted the therapeutic/prophylactic potential of engineered EVs loaded with QCT against SARS-CoV-2 infection. Several biotechnological engineering approaches are available to deliver EVs loaded with QCT nanoparticles. Among these biotechnological advances, a specific approach with significantly higher efficiency and yield has to be opted to fabricate such drug delivery of nano molecules, especially to combat SARS-CoV-2 infection. The current treatment regime protects the human body from virus infection but has some limitations including drugs and long-term steroid side effects. However, the vaccine strategy is somehow effective in inhibiting the spread of coronavirus disease-19 (COVID-19) infection. Moreover, the proposed exosomal therapy met the current need to repair the damaged tissue along with inhibition of COVID-19-associated complications at the tissue level. These scientific findings expand the possibilities and predictability of developing a novel and cost-effective therapeutic approach that combines the dual molecule, EVs and QCT nanoparticles, to treat SARS-CoV-2 infection. Therefore, the most suitable engineering method to fabricate such a drug delivery system should be better understood before developing novel therapeutics for clinical purposes.

## 1 Introduction

QCT is a plant flavonoid, also known as 3,3′,4′,5,7-pentahydroxyflavone, and is present in naturally occurring vegetables, grains, leaves, and seeds in the form of QCT glycosides bounded with residual sugars (1). QCT exhibits various biological properties, including antioxidant (2), immunoprotective (3), anti-inflammatory, and anti-viral (4). QCT, being a promising anti-viral, showed its effect by inhibition of biological enzymes including protease (5), polymerase (6), reverse transcriptase (7), DNA gyrase and proteins of viral capsids (8, 9). Furthermore, QCT functions as a protein kinase inhibitor, a phytoestrogen, a quinone, a chelator, a free radical scavenger, and an Aurora kinase inhibitor (10). Previously published studies showed that neutrophils upon treatment with QCT flavonoids demonstrated suppression of pro-inflammatory gene mRNA along with mi-RNA modulation (11, 12). QCT application in COVID-19 infection occurs significant therapeutic effect if used in combination with standard care of treatment. Moreover, recently two studies demonstrated effectiveness of using QCT with remdesivir and favipiravir in hospitalized patients with severe SARS-CoV-2 infection. The patients were given 1000 mg QCT along with anti-viral drugs daily and found to reduced serum levels of ALP, q-CRP, and LDH in the intervention group compared to those who were on only standard care of treatment (13). Similarly in another randomized, open-label, and controlled clinical study, the add-on supplement of the QCT particles with standard care of treatment showed viral clearance within 1 week of the infection (14). Such studies favoured the utility of using QCT nanoparticles either alone or in combination with standard care of treatment for effective management and treatment of COVID-19 infection.

QCT showed its abundance in many plants, including apples, grapes, green tea, citrus fruits, cherries, onions, coffee, red wine, and others (15). However, QCT exhibits low bioavailability and hence the need to supplement with other supplements such as catechins, resveratrol, and genistein to increase the high absorption within the intestinal cells (16–19). Moreover, QCT has several limitations in pharmaceuticals, including instability, low solubility, poor permeability, and low bioavailability. Within the past decades, numerous nanotechnology-based approaches were designed to combat such limitations. Some of the delivery approaches of QCT to overcome such limitations include liposomes, inclusion complexes, micelles, and nanoparticles. These nanotechnology-based approaches have proved beneficial in the treatment of several human diseases, including severe acute respiratory syndrome coronavirus 2 (SARS-CoV-2) infections.

**Figure 1:**
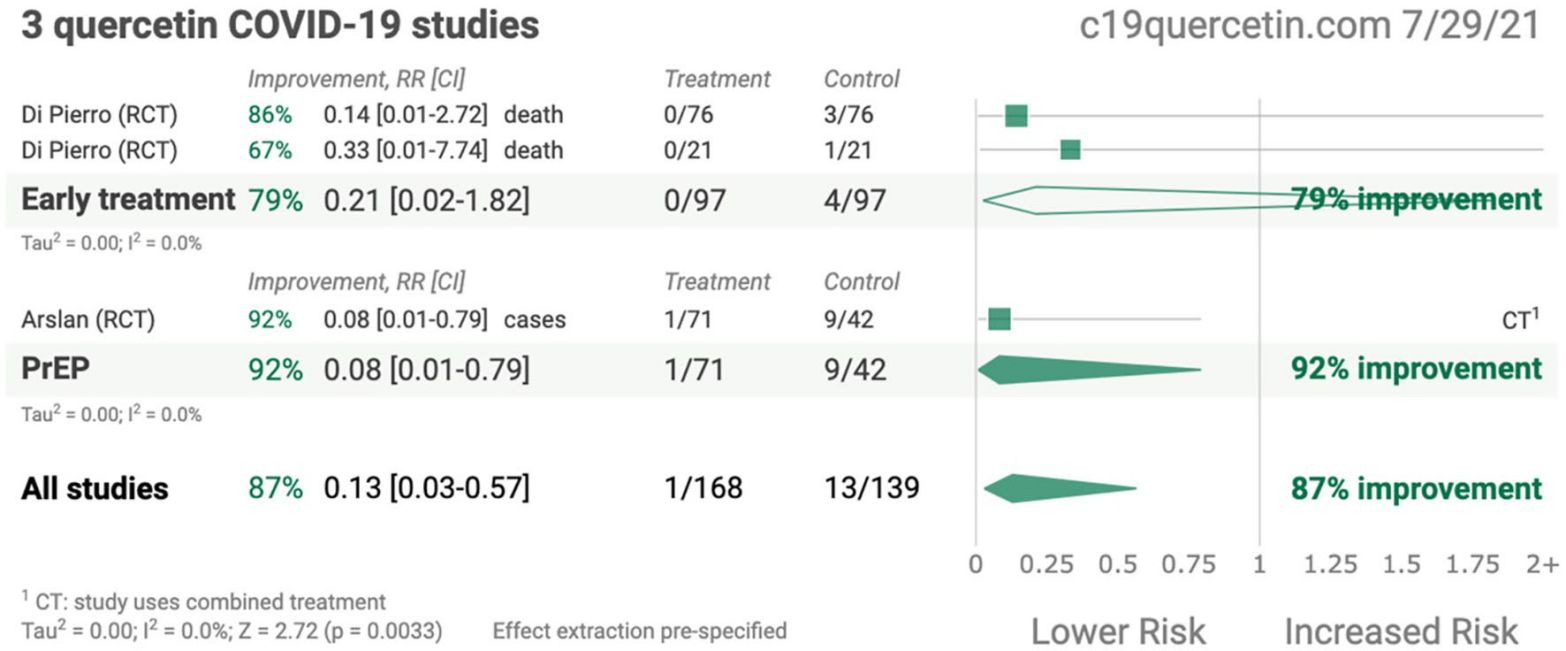
Summary of quercetin COVID-19 studies (Adopted under Creative Commons Attribution-Non Commercial v4.0 License from Ref. 24).

QCT possessed anti-viral characteristics and thereby hampered the life cycle of SARS-CoV-2 by interfering with viral replication cycles and lowering the inflammation responses. Recently published studies demonstrated that QCT interferes with the viral replication cycle of SARS-CoV-2 and thereby offers numerous opportunities to explore QCT as a supplement in the form of nanoparticles as therapeutics in COVID-19 infection (20–22). A recent study also claimed that QCT supplementation offers 87% improvement in the form of survival outcomes in patients with SARS-CoV-2 infection and may be more beneficial during the early stages of COVID-19 infection (21–24). In another randomized controlled trial (RCT), QCT showed prophylaxis among COVID-19- infected patients (25) Some of the pioneer studies related to QCT therapy in COVID-19 infection are presented in **Figure 1** (26).

It is still a debatable matter that vaccines are associated with a risk of local and systemic inflammatory immune responses along with systemic toxicity, and hence supplementation of QCT nanoparticles may mitigate such effects. The present study evaluates the characteristics of QCT nanoparticles and extracellular vesicle-based delivery along with the immunomodulation mechanism of these nanoparticles in COVID-19 infection.

## 2 QCT chemistry

**Figure 2:**
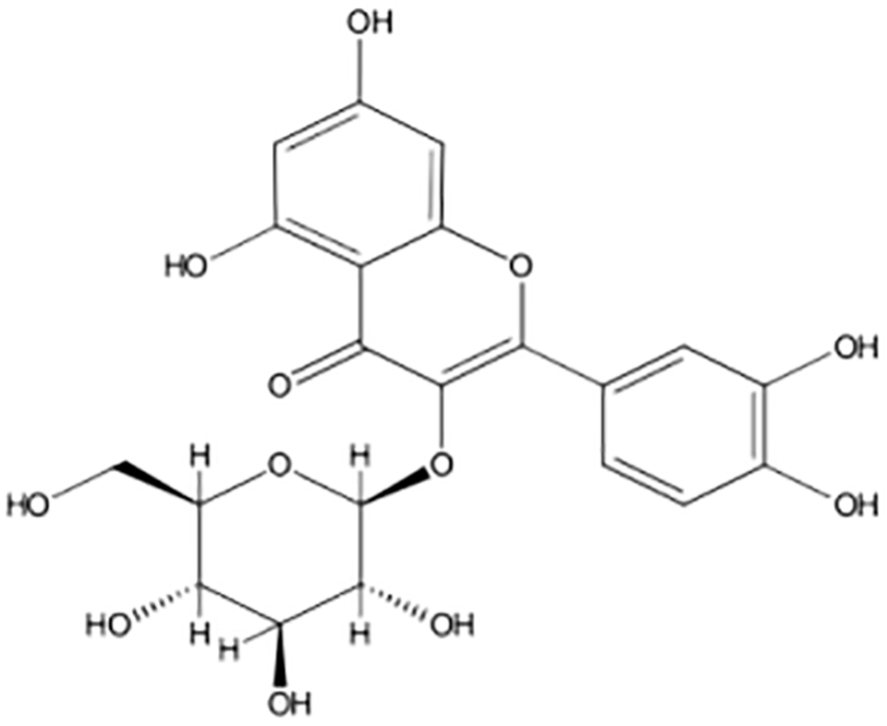
2D structure of quercetin.

The chemical name of QCT is C_15_H_10_O_7_ or 2-(3,4-dihydroxyphenyl)-3,5,7-trihydroxychromen-4-one or 3,3,4,5,7-pentahydroxyflavone, which tends to present conjugation with glucose, xylose, and rutinose *via* hydroxyl (OH) functional groups present in its structure, which imparts the antioxidant tendency because it is composed of five hydroxyl groups as demonstrated in **Figure 2**. QCT is of nutritional importance and is majorly present in the form of QCT-3-O-glycoside rather than aglycones (27–31). Substitution of OH functional groups at positions C3, C5, and C7, as well as H atom substitution at C3’ and OCH_3_ substitution at C4’, proved to be beneficial and protective to cells (32). Furthermore, QCT has a chemical composition that includes 3′, 4′-OH groups (B ring) and 2, 3-double bond conjugation with a 4-oxo functional group present in the C ring, which helps to reduce oxidative stress (33). Another study found that the orthodihydroxy, 4-carbonyl, and 3′, 4-OH group substitutions on the B, C, and A-rings, respectively, exhibit metal ion chelator activity of QCT (34).

## 3 Characteristics of QCT

QCT has significant solubility in lipids and alcohols but shows poor solubility in water. A previous study observed that QCT glycoside has a high affinity for water due to the presence of several glycosyl functional groups (35) (**Table 1**). QCT is usually present in two forms, namely glycosides and glycones, and follows passive diffusion and an anion-transporting pathway for absorption into the small intestine. QCT metabolism occurs in the liver, intestines, and kidneys with a half-life ranging from 11 to 28 h and an average terminal half-life of 3.5 h (36, 37). A study showed that QCT glucoside showed better solubility than rutinosides in the small intestine upon hydrolysis by glucosidases (38, 39). The enzymes of the intestinal mucosa and epithelial cells transform QCT and its derivatives into diverse metabolites, including phenolic acids. These metabolites are excreted majorly by the kidney through urine in the form of benzoic acids (40).

**Table 1:** Chemical and Physical properties of quercetin (Adopted from Ref 26).

Property Name	Property Value	Reference
Molecular Weight	302.23	Computed by PubChem 2.1 (PubChem release 2021.05.07)
XLogP3	1.5	Computed by XLogP3 3.0 (PubChem release 2021.05.07)
Hydrogen Bond Donor Count	5	Computed by Cactvs 3.4.8.18 (PubChem release 2021.05.07)
Hydrogen Bond Acceptor Count	7	Computed by Cactvs 3.4.8.18 (PubChem release 2021.05.07)
Rotatable Bond Count	1	Computed by Cactvs 3.4.8.18 (PubChem release 2021.05.07)
Exact Mass	302.04265265	Computed by PubChem 2.1 (PubChem release 2021.05.07)
Monoisotopic Mass	302.04265265	Computed by PubChem 2.1 (PubChem release 2021.05.07)
Topological Polar Surface Area	127 Å²	Computed by Cactvs 3.4.8.18 (PubChem release 2021.05.07)
Heavy Atom Count	22	Computed by PubChem
Formal Charge	0	Computed by PubChem
Complexity	488	Computed by Cactvs 3.4.8.18 (PubChem release 2021.05.07)
Isotope Atom Count	0	Computed by PubChem
Defined Atom Stereocenter Count	0	Computed by PubChem
Undefined Atom Stereocenter Count	0	Computed by PubChem
Defined Bond Stereocenter Count	0	Computed by PubChem
Undefined Bond Stereocenter Count	0	Computed by PubChem
Covalently-Bonded Unit Count	1	Computed by PubChem
Compound Is Canonicalized	Yes	Computed by PubChem (release 2021.05.07)

## 4 QCT nanoparticles as therapeutics

**Figure 3:**
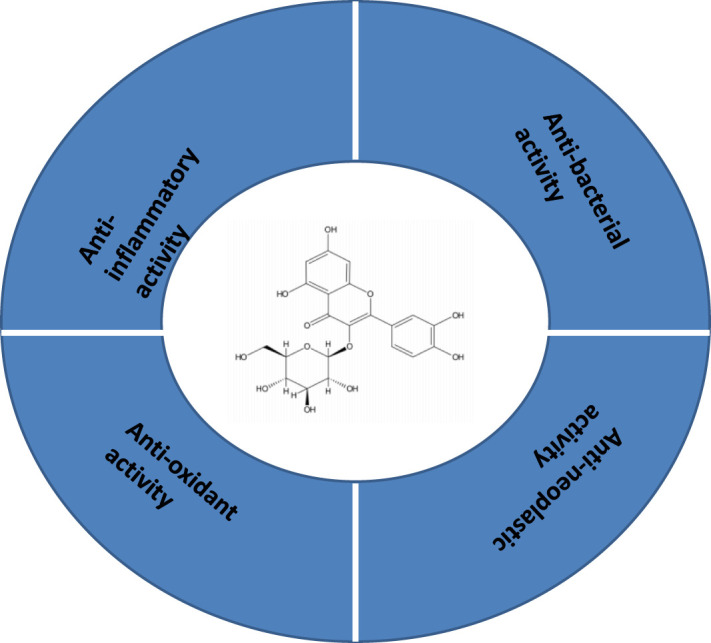
Structure and therapeutic application of quercetin.

**Figure 4:**
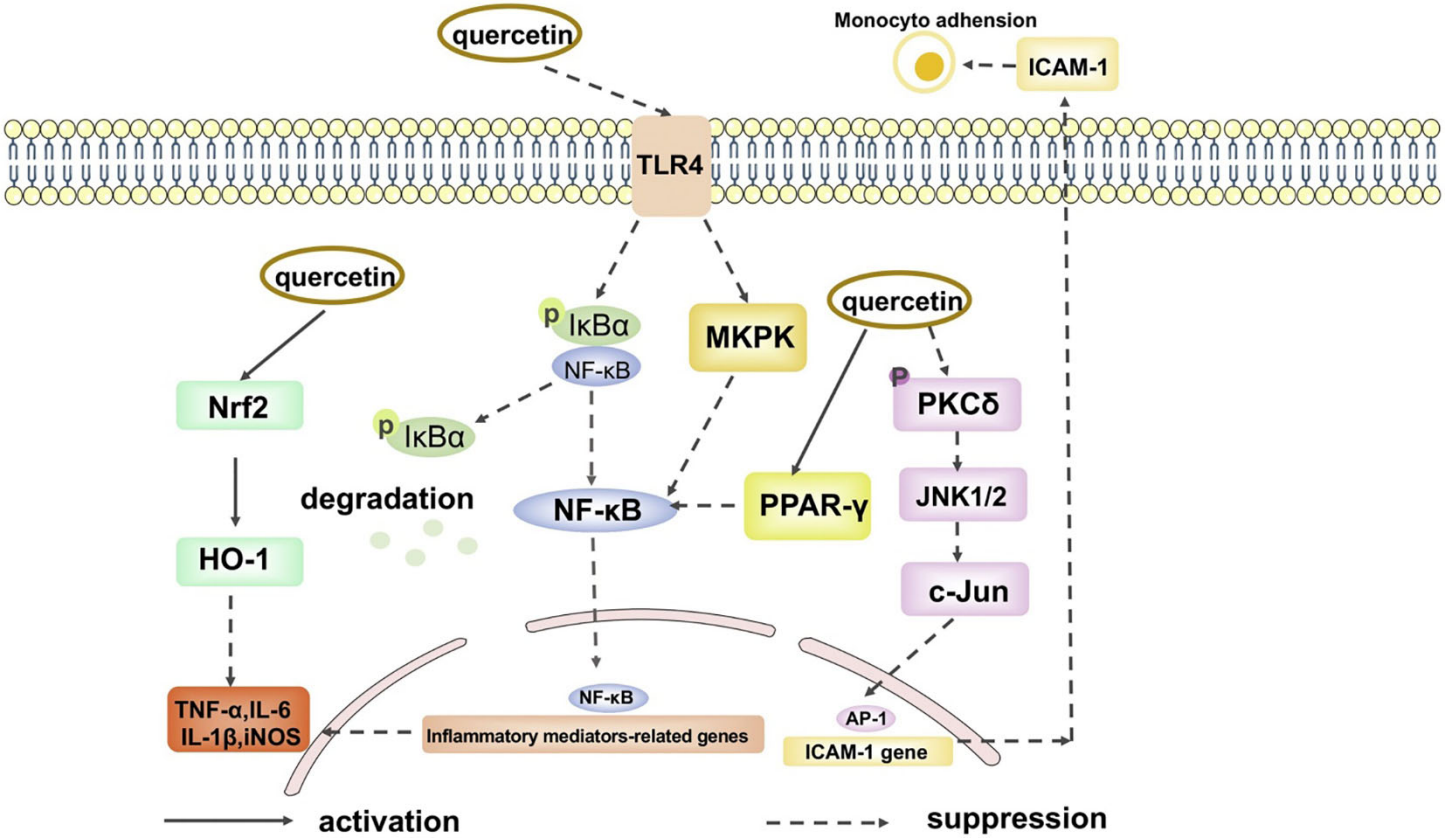
The anti-inflammation signal pathways of quercetin. (Adopted under Creative Commons Attribution-Non-Commercial v4.0 License from Ref. 39).

Fabrication of nanoparticle-based drug delivery systems has been validated and tested in several pre-clinical and clinical trials for the treatment and diagnosis of several diseases in the past decades (**Table 2**). The therapeutic potential of QCT nanoparticles includes antioxidant, anti-inflammatory, anti-bacterial and anti-neoplastic activities (**Figures 3**, **4**) (41). These nanoparticle delivery agents such as polymeric micelles, liposomes, quantum dots, chitosan, Polylactic/glycolic acid (PLGA) and PLGA-based nanoparticles, polymeric micelles, dendrimers, and inorganic nanoparticles have been explored well for targeted delivery of desired drugs, proteins, peptides, and nucleic acids (42). Entrapment of the desired drug into the vicinity of nanoparticles enhances its bioavailability and stability with prolonged circulation time, which helps in improving therapeutic outcomes (43). These nanoparticles enable drug delivery by exhibiting functional and structural modifications like ligand linkage within a single delivery partner.

**Table 2:** List showing clinical trials using quercetin. (Adopted from Ref 26).

#	Linked Compound CID	CTID	Title	Phase	Status	Date
1	**30623165280343**	NCT04785300	ALSENLITE: Senolytics for Alzheimer’s Disease	Phase 1/Phase 2	Enrolling by invitation	2022-05-12
2	**5280343**	NCT05371340	QCT’s Effect on Bone Health and Inflammatory Markers	N/A	Completed	2022-05-12
3	**30623165280343**	NCT02652052	Hematopoietic Stem Cell Transplant Survivors Study (HTSS Study)	N/A	Recruiting	2022-04-15
4	**5280343**	NCT04907253	Quercetin in Coronary Artery By-pass Surgery	Phase 2	Recruiting	2022-04-05
5	**5280343**	NCT03476330	Quercetin Chemoprevention for Squamous Cell Carcinoma in Patients With Fanconi Anemia	Phase 2	Recruiting	2022-02-02
6	**30623165280343**	NCT04685590	Senolytic Therapy to Modulate the Progression of Alzheimer’s Disease (SToMP-AD) Study	Phase 2	Recruiting	2022-01-24
7	**5280343**	NCT01720147	Quercetin in Children With Fanconi Anemia; a Pilot Study	Phase 1	Active, not recruiting	2022-01-18
8	**5280343**	NCT04258410	Quercetin for Cardio-Skeletal Muscle Health and Estrogen Deficiency	Phase 4	Suspended	2021-12-03
9	**5280343**	NCT03989271	Biological Effects of quercetin in COPD	Phase 1/Phase 2	Recruiting	2021-10-06
10	**5280343**	NCT05037240	Quercetin in the Prevention of Covid-19 Infection	N/A	Completed	2021-09-08
11	**5280343**	NCT04853199	Quercetin In The Treatment Of SARS-COV 2	Early Phase 1	Recruiting	2021-07-28
12	**5280343**	NCT04851821	The Effectiveness of Phytotherapy in SARS-COV2(COVID-19)	Phase 1	Completed	2021-07-27
13	**306231652803435281614**	NCT04313634	Targeting Cellular Senescence With Senolytics to Improve Skeletal Health in Older Humans	Phase 2	Recruiting	2021-07-21
14	**650645280343**	NCT01912820	Effect of quercetin on Green Tea Polyphenol Uptake in Prostate Tissue From Patients With Prostate Cancer Undergoing Surgery	Phase 1	Completed	2021-06-23
15	**30623165280343**	NCT04063124	Senolytic Therapy to Modulate Progression of Alzheimer’s Disease	Phase 1/Phase 2	Recruiting	2021-06-21
16	**946528034341004091**	NCT02463357	Three New Ideas to Protect Special Forces From the Stress of High Altitude	Phase 4	Completed	2021-03-30
17	**2399474981710528034354670067**	NCT04468139	The Study of Quadruple Therapy Zinc, quercetin, Bromelain and Vitamin C on the Clinical Outcomes of Patients Infected With COVID-19	Phase 4	Recruiting	2020-07-13
18	**30623165280343**	NCT02874989	Targeting Pro-Inflammatory Cells in Idiopathic Pulmonary Fibrosis: a Human Trial	Phase 1	Completed	2020-05-12
19	**5280343**	NCT03943459	Sirtuin-1 and Advanced Glycation End-products in Postmenopausal Women With Coronary Disease	Phase 3	Recruiting	2019-10-04
20	**5280343**	NCT02989129	Trial of quercetin in the Treatment and Prevention of Chemotherapy-induced Neuropathic Pain in Cancer Patients	Early Phase 1	Withdrawn	2018-04-18
21	**5280343**	NCT00402623	The Effect of quercetin in Sarcoidosis	N/A	Completed	2017-02-24
22	**5280343**	NCT01708278	Beneficial Effects of quercetin in Chronic Obstructive Pulmonary Disease (COPD)	Phase 1	Completed	2016-12-26
23	**5280343**	NCT02226484	Can quercetin Increase Claudin-4 and Improve Esophageal Barrier Function in GERD?	Phase 1	Completed	2016-12-20
24	**5280343**	NCT01438320	Q-Trial in Patients With Hepatitis C	Phase 1	Completed	2015-03-20
25	**7131270418397995448913664177412901002452803434454211346946484432690122172882444254441184**	NCT01839344	Effects of quercetin on Blood Sugar and Blood Vessel Function in Type 2 Diabetes.	Phase 2	Completed	2015-03-18
26	**5280343**	NCT00913081	Advancing Niacin by Inhibiting Flushing (ANTI-FLUSH)	Phase 4	Completed	2015-03-05
27	**722765280343**	NCT01691404	Study on the Effects of Epicatechin and QCT Supplementation on Vascular Function and Blood Pressure (FLAVO)	N/A	Completed	2013-04-23
28	**5280343**	NCT01732393	Effect of QCT in Prevention and Treatment of Oral Mucositis	Phase 1/Phase 2	Completed	2012-12-05
29	**5280343**	NCT01375101	Therapeutic Effect of quercetin and the Current Treatment of Erosive and Atrophic Oral Lichen Planus	Phase 1	Unknown status	2011-07-26
30	**154888752803439695165280805**	NCT00003365	Sulindac and Plant Compounds in Preventing Colon Cancer	N/A	Terminated	2011-01-27
31	**5280343**	NCT01168739	Effect of Combined Exercise, Heat, and quercetin Supplementation on Whole Body Stress Response	N/A	Completed	2010-07-23

N/A: Not Applicable.

Moreover, several nanoparticles, including liposomes, offer opportunities to deliver hydrophobic drug molecules like QCT to explore its improved encapsulation efficiency, targeted delivery with extended circulation time within the body with controlled release, and high therapeutic efficiency. In the past decades, significant advances have been made in developing engineered nanoparticles containing QCT as therapeutics. To achieve this, researchers have explored liposomes, PLGA nanoparticles, polymeric micelles, metal-organic frameworks (MOFS), inorganic molecules, and other delivery systems for biomacromolecule-based nanoparticles. Some limitations associated with these nanoparticles are systemic toxicity and adverse effects, and fewer tendencies to cross the blood-brain barrier (BBB). To combat such limitations, EVs have been a preferred choice nowadays for delivering nanoparticles, including QCT.

## 5 EVs-based QCT nanoparticles as therapeutics

**Figure 5:**
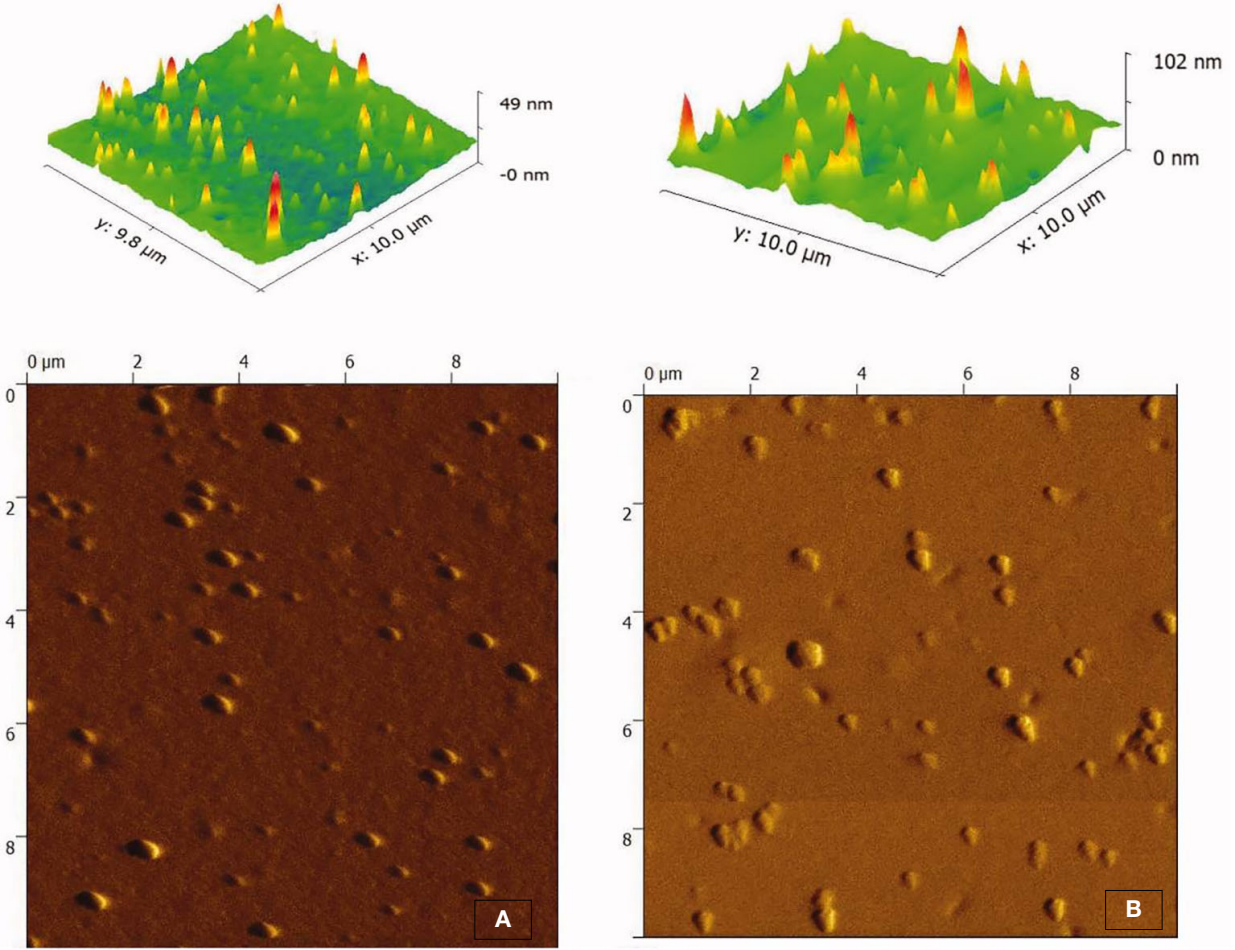
Morphology determination by atomic force microscopy **(A)** Exosome and **(B)** Exosome loaded with quercetin. (Adopted under Creative Commons Attribution-Non-Commercial v4.0 License from Ref. 43).

Extracellular vesicles (EVs) are nano-sized lipid bilayer vesicles secreted by metabolically active cells that have the potential for functional and structural modifications for drug delivery to targeted organs. Compared to other delivery agents, EVs offer several advantages, including biocompatibility, negligible systemic toxicity and adverse effects, increased bio-distribution, and high transmission efficiency with a tendency to deliver biomolecules including proteins, peptides, lipids, and nucleic acids (44). Previously, EVs demonstrated promising potential for delivering a wide range of drugs and biomolecules as carriers with significantly improved bioavailability and high transmission across the BBB. A study showed plasma-derived exosome-loaded QCT nanoparticles showed improved bioavailability with therapeutic effect in Alzheimer’s disease by inhibiting cyclin-dependent kinase 5 (CDK5) facilitated phosphorylation of protein Tau (45). The atomic force microscopic image of exosome loaded QCT was shown in **Figure 5**. In another study, authors engineered exosomes containing QCT nanoparticles and monoclonal antibodies against GAP43 (mAb GAP43) and found a therapeutic effect in cerebral ischemia by decreasing reactive oxygen species (ROS) (46).

**Figure 6:**
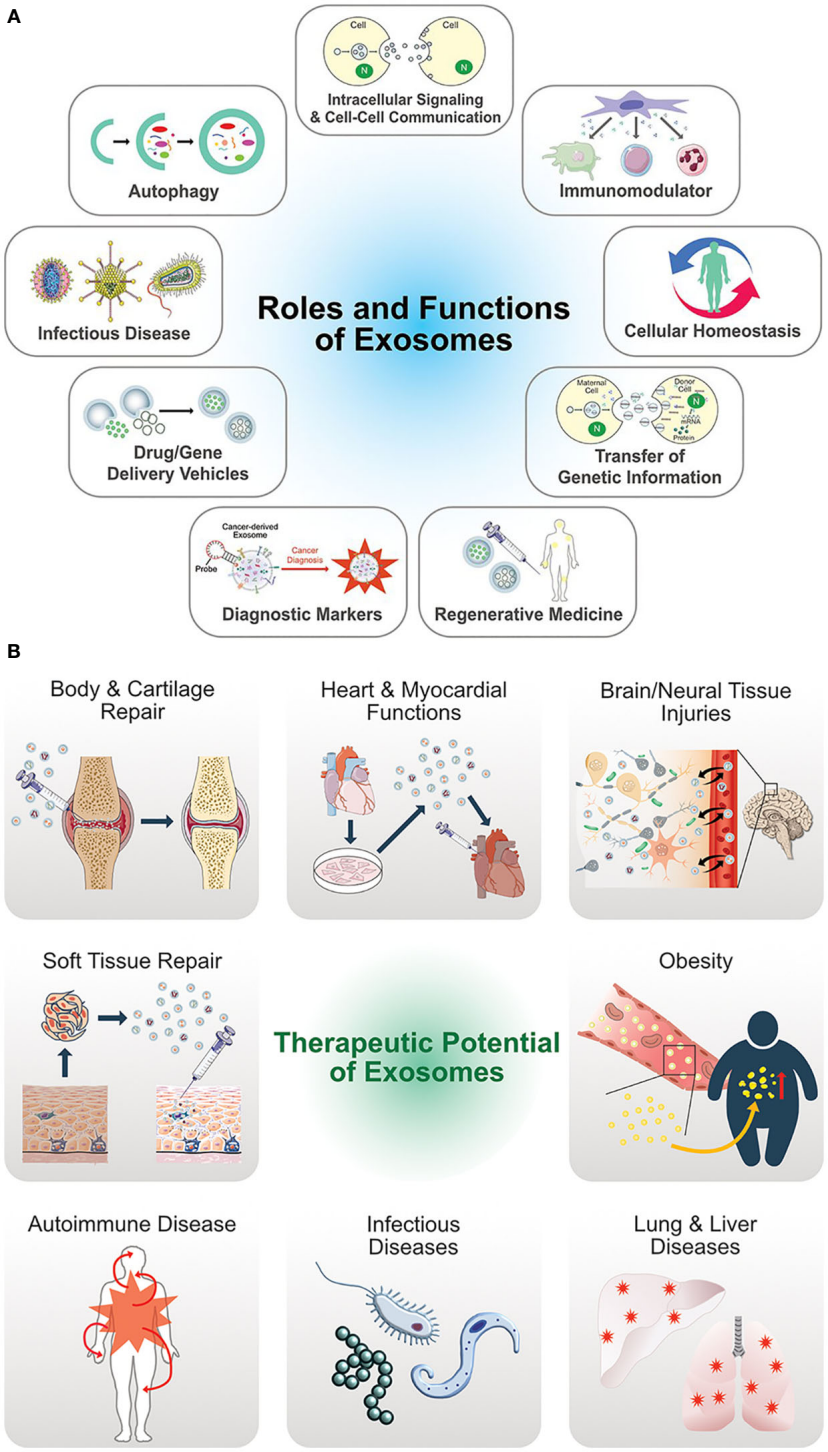
**(A)** Multifunctional aspects biological functions of exosomes. **(B)** Therapeutic potential and versatile clinical implications of exosomes. (Adopted under Creative Commons Attribution-Non-Commercial v3.0 License from Ref. 46).

The authors of a recently published study (47) assessed the nutraceutical properties of EVs containing QCT and saponin. Fabricated EVs loaded with QCT and saponins extracted from black bean extract (*Phaseolus vulgaris* L.) along with three more phytochemicals at a single time to deliver them all at once to the target site or recipient cells. The study concluded that EVs loaded with nanoparticles have increased bioactivity compared to the phytochemicals used alone with EVs (47). Above, studies favor the development of new products containing EVs containing nutraceuticals for the treatment of several diseases as EVs serve various therapeutic functions and roles (48) (**Figure 6**).

## 6 EVs modification approaches

EV modification and bioengineering have gained increased attention in recent years. Due to their ability to manipulate for delivering multiple biological molecules such as proteins, drugs, lipids, and nucleic acids (coding or non-coding), studies have demonstrated the beneficial role of EVs as drug carriers, proving their therapeutic role in several diseases (49). Previously published literature supported our study as they claimed that EVs exhibits immunomodulation characteristics and hence can be effectively used in the treatment of lungs affected with SARS-CoV-2 infection (48). Moreover, EVs also have therapeutics role in the treatment of cardiovascular diseases associated with COVID-19 by repairing ischemic myocardial injury by inducing neovascularization (49). Moreover, study also claimed protective role of EVs against acute kidney injury and acute liver injury (48). The modification approach of EVs is further classified into two types: I. direct modification and II. indirect modifications. The direct modification approach refers to EV engineering that is further classified into physical and chemical modifications (50).

### 6.1 Physical engineering

EVs membrane and indigenous content can be modified using different physical approaches which are mentioned below;

#### 6.1.1 Engineering with EVs surface

Conventional surface modifications of EVs exploit the attachment of proteins, lipids, and polypeptides on their outer membrane using specific methods. Such EV modification was found to be beneficial in the treatment of leukaemia and tumor growth suppression (51). Because EVs are composed of a lipid bilayer, they can be easily conjugated or modified using liposomes, as demonstrated by previous studies (52, 53). In one of the previously published studies, authors fused exosomes derived from bone marrow mesenchymal stromal cells (BMSCs) with liposomes containing polypyrrole nanoparticles for the treatment of diabetic peripheral neuropathy (53). Moreover, liposomes were fused with the MSC-derived EVs to incorporate miR-34a and this system was tested against breast cancer progression (54).

Several studies were conducted on EV surface modification and demonstrated that the linkage of glycosyl phosphatidylinositol (GPI) on the EV surface imparts stability to deliverable EVs and also protects such EVs from hydrolytic degradation (55, 56). Another study on the SARS-CoV-2 virus discovered that EVs contain the enzyme ACE-2, and their levels can be estimated using protein palmitoylation (57). Two enzyme systems, namely zinc finger DHHC-Type Palmitoyl transferase 3 (ZDHHC_3_) and acyl protein thioesterase 1 (LYPLA1), performed the mechanism of palmitoylation and de-palmitoylation of such proteins. The authors fabricated engineered EVs with S-palmitoylation targeted sequences as a therapeutic and prophylactic approach against SARS-CoV-2 and thereby protect the lungs from inflammation (57).

#### 6.1.2 Engineering with EVs content

The efficiency of the EVs for targeted drug delivery can also be fairly improved by doing engineering with EVs content through specific approaches including incubation, sonication, electroporation, and others that are helpful in loading many drugs, proteins, peptides, miRNAs and long-non-coding RNAs into the EVs as also suggested by previously published study (50, 58).

##### 6.1.2.1 Incubation

This engineering method involves the simple incubation of EVs with the desired drug or biological molecules, which is still known as “passive loading of cargo.” The driving force behind the loading of desired cargo in the EVs is the interplay between the concentration of drugs inside and outside the EV medium that allows the transfer of drugs or desired cargo within the lipid bilayer of the EVs. Some studies implicated the incubation approach for loading anti-cancerous drugs including paclitaxel and doxorubicin into the EVs and achieved significant chemotherapeutic effects (59, 60). In another study, the authors used this method for loading curcumin nanoparticles into the EVs and evaluated its effect as an anti-inflammatory approach (61). This is a cost-effective and simple method that does not require high throughput facilities but with the major limitation of low yield and transport of hydrophobic cargoes only.

##### 6.1.2.2 Sonication

This engineering approach relies on sound waves for the generation of mild shearing forces that are needed for the disruption of lipid bilayers of EVs so that desired drug or biological molecules mediate transport into them. Kim and their coworkers successfully loaded paclitaxel and doxorubicin into the EVs and exploited them for cancer treatment (62). It is reported that the sonication approach decreases the viscosity of the EVs membrane and thereby allows the passage of cargo inside it (62). This method exhibits high loading efficiency compared to the simple incubation approach, with some limitations in shearing forces that are not suitable to deliver labile biological molecules such as miRNAs. This approach resulted in improved drug loading efficiency with better biocompatibility and controlled release of desired drugs with incubation and electroporation (63).

##### 6.1.2.3 Electroporation

This engineering approach is a commonly used method for EV modification using the electric field for cargo loading. An electric field disrupts the lipid bilayer of the EVs, thereby facilitating the entry of the desired drug or biomolecule inside it. This method is most suitable for loading hydrophilic cargo like miRNA, siRNA, and other nucleic acids (64, 65). Johnsen et al. reported that electroporation tends to aggregate the EVs without altering their functions (66). For maintaining the structural conformation of the EVs, the authors used trehalos-containing buffer during the electroporation (66). Another study used electroporation for the loading of doxorubicin drug with hydrophobically modified miRNA 159 into the EVs for the treatment of tumors (67). This approach, however, facilitates the rapid entry of the desired drug into the EVs but simultaneously damages the structural integrity of such EVs, thereby reducing the efficiency of drug/molecule loading (68–70). Furthermore, one study used electroporation to load miRNA 155 into the EVs with good efficiency at a voltage of 0.13–0.2 kV (concentration of EVs 500–1000 mg/mL) (71).

##### 6.1.2.4 Freeze thawing

This engineering approach involves the temporary generation of pores on the EVs’ membrane upon multiple freezing and subsequent thawing cycles that facilitate the entry of drugs inside EVs. A freeze cycle consists of -80^0^C while thawing at 37^0^C, repeated several times. Some studies have reported the formation of aggregates following this approach while loading drugs into EVs (72, 73). Hanley and their co-workers successfully loaded the enzyme catalase into the EVs using this approach (72). This approach offers the benefit of mass production of engineered EVs compared to the ultrasonication method with improved drug loading efficiency (62, 74). Moreover, freeze-thaw process is associated with some disadvantages as evident by the previously published study (A). The repeated process of the freeze-thaw severely affected the membrane stability of the EVs (75). Author of another study demonstrated the decrease in EVs concentration up to 2 folds even after the single freeze-thaw cycle and also reported changes in the structural morphology of EVs post storage (75).

##### 6.1.2.5 Extrusion

This engineering approach involves the use of a small-sized polycarbonate porous membrane for reversible disruption of the phospholipid bilayer of the EVs to facilitate the entry of desired drugs/cargo inside the EVs (72, 73). This method results in the production of uniform-sized EVs with efficient drug loading compared to incubation and electroporation under controlled conditions (74). Extrusion, however disrupt the membrane integrity of the EVs thereby causing leakage of loaded drugs. During the process of extrusion, the EVs membrane faces vigorous forces and as a resultant, disruption occurs. This is also evident with the changes in the membrane zeta potential and membrane protein disorganization. Fuhrmann and their coworkers proved that EVs prepared using the extrusion method exhibits cytotoxicity compared to non-extruded EVs (76). The authors compared the loading of siRNA into EVs using the natural mode of EV secretion from cells with EV mimics prepared by multiple sequential extrusions of MCF10A cells and discovered that EV mimics had the highest drug loading (77, 78).

### 6.2 Chemical engineering

Chemical-based modification or engineering of EVs refers to the transformation of EVs’ surfaces. This can be further classified as covalent (reaction between EVs and chemical linkers) and non-covalent (electrostatic interaction) based modifications.

#### 6.2.1 Covalent-based modification of EVs

Covalent modification, or click chemistry, involves the chemical conjugation of ligands with EV surfaces. Several amino acids can be effectively conjugated on EV surfaces through azide-alkyne cyclo-addition click chemistry (79, 80). Another study fabricated azide-labeled c-RGD peptide on an EV surface using alkyne-azide cyclo-addition with dibenzo cyclooctyne to treat ischemic brain injury (81). The Click chemistry-based modification exhibits high specificity, selectivity, and high compatibility without affecting the structural and functional integrity of the EVs.

#### 6.2.2 Non-covalent based modifications of EVs.

Non-covalent-based EVs’ modification mostly involves electrostatic and ligand-based alteration to EVs. The presence of a lipid bilayer on EVs’ surfaces imparts a negative charge with a zeta potential of approximately-8.82 mV, making them suitable to add cations on their surfaces (82). Authors have fabricated EVs-based immune blockers to promote the phagocytosis of tumor cells by macrophages through a non-covalent-based modification approach (83, 84). However, ligand-receptor-based modification of EVs involves hydrophobic ligand interactions on the lipid component present on the surface of EVs. For therapeutic and diagnostic purposes, liposomes modified using polyethylene glycol (PEG) is commonly used for EV modification to load a desired molecule of interest.

### 6.3 Indirect engineering approach for modification of EVs

Most of the cells secrete EVs in the medium that can be further exploited for therapeutic and diagnostic purposes. The EV-producing parental cells can be genetically and metabolically engineered to produce modified EVs with enhanced drug-loading capacity.

#### 6.3.1 Genetic engineering approach for EVs modification

Genetic engineering approaches for the modification of EVs producing parental cells to achieve specific and targeted drug/biomolecule loaded EVs have become sophisticated within the past decades. Membrane proteins can be efficiently linked with EVs using this method. This involves transfection (via viral or non-viral invasion/infection) of the EVs producing parental cells using specific gene targets that allow the manufacturing of cargo-loaded EVs during the biogenesis process. Jiang et al., reported two types of viral vectors for modification of EVs using a genetic engineering approach, i.e., retroviral and adenoviral, for delivery of tumor necrosis factor (TNF)-stimulated gene-6 (TSG-6) (85). A study reported use of MSCs-derived modified EVs using a genetic engineering approach with miRNAs delivery (86). In another study, authors fabricated engineered EVs expressing Lamp2b conjugated integrin-specific iRGD peptide for the treatment of breast cancer (87). Similarly, in another study, engineered EVs were synthesized derived from HEK293T cells expressing Lamp2b conjugated with IL-3 fragments for the treatment of chronic myeloid leukaemia (CML) (88).

Rivoltini and their co-workers transfected the K562 cell line with lentiviral human membrane TRAIL (TNF-Related Apoptosis-Inducing Ligand) for fabrication of TRAIL (+) EVs for apoptosis of cancerous cells (89). In another study, dendritic cells (DCs) were transfected using a genetic engineering approach with a Lamp2b-modified pEGFP-C1 vector to produce RVG-modified EVs (90). The major limitation associated with this approach is that it affects the efficiency of the drug/molecule loading to EVs loading of foreign impurities that result in a decrease in EV purity.

#### 6.3.2 Metabolic engineering approach for EVs modification

The metabolic engineering approach for modification of EVs delivers metabolites or biological molecules including amino acids, lipids, and sugar moieties to the growth medium of parental cells for promotion of biosynthesis. These biomolecules can be integrated into the proteome, liposomes, and glycoproteins present in the EVs (91). This engineering approach used click conjugation to attach glycans and glycoproteins with chemically active azide functional groups and bio-orthogonal moieties to produce metabolically engineered EVs (92). In another study, authors replaced the naturally occurring methionine amino acid with L-azidohomoalanine (AHA), an azide-bearing amino acid (which is an analogue of natural methionine) inside EVs (93). In continuation with this study, the introduction of AHA into the exosomes offers an additional azide active site for bio-conjugation, thereby providing an additional free site for binding of desired cargo/molecule of interest for therapeutic and diagnostic purposes (94).

## 7 Mechanism of action of EVs loaded QCT nanoparticles in SARS-CoV-2

The spread of severe acute respiratory syndrome coronavirus-2 (SARS-CoV-2) is a coronavirus disease (COVID-19) progressing as a worldwide pandemic targeting the lungs of infected patients. The SARS-CoV-2 virus is an RNA virus that recognizes human cells through angiotensin-converting enzyme 2 receptors (ACE-2) present on the epithelial cells for its entry (95–97). Virus infection triggers inflammatory responses such as cytokine storm, oxidative stress, and acute respiratory distress syndrome (98, 99). Studies demonstrated the anti-SARS-CoV-2 activity of QCT nanoparticles by inhibiting the binding of viruses with ACE-2 receptors, lowering the pro-inflammatory cytokines, and down-regulating the expression of the RdRp gene (100–102).

Docking studies conducted recently showed the anti-SARS-CoV2 effect of QCT molecules (101). The study showed that QCT inhibits the expression of the NLRP3 inflammasome through various regulatory proteins (101). The study also concluded that QCT nanoparticles can be prospectively performed as antioxidant, anti-inflammatory, and analgesic characteristics and, thereby, can be used to treat severe inflammation associated with SARS-COV-2 infection in COVID-19 (101).

Studies demonstrated that QCT inhibits the expression of the NLRP3 inflammasome mediated through TXNIP (103). In an animal study conducted on a spinal cord injury model, QCT nanoparticles demonstrated anti-inflammatory and antioxidant properties along with inhibition of pro-inflammatory cytokine generation (103). A study quoted said QCT showed antioxidant and anti-inflammatory roles that further protected the cells from apoptosis (103). In-silico finding of SARS-CoV-2 protease proteins (PDB ID: 6LU7) showed an inhibitory effect of QCT by forming new hydrogen bonds with some amino acid residues (His164, Glu166, Asp187, Gln192, and Thr190) of 6LU7 (100, 102). Moreover, another preclinical study of a mouse model conducted on asthma showed that QCT therapy lowers the count of white blood cells (WBCs) along with eosinophils in the blood, lung parenchyma, and bronchoalveolar lavage fluid (104). Previously published study showed that, QCT particles mediate inhibition of p38 mitogen-activated protein kinase (MAPK) and NF-k (105). Another author demonstrated that QCT nanoparticles downregulate the expression of histamine, prostaglandin D2, leukotriene, and granulocyte-macrophage colony-stimulating factors (106). In one of the recently published study, the authors have reported the role of quercetin as antituberculosis, antioxidant and cytotoxicity and therefore favored this study (107).

Several studies focused on the immune-modulatory and anti-inflammatory roles of MSC-derived EVs similar to their parental cell source (108, 109). In a preclinical study, MSC-derived EVs showed therapeutic effects in Acute Respiratory Distress Syndrome (ARDS) (110). Another study showed that EVs initiate anti-inflammatory responses that further reduce the severity of lung injury through the maintenance of alveolar epithelium (111, 112). It has been seen from previous literature that the use of EVs or nano decoys slows down the progression of viral infection through binding with viral cells (113–115). Studies also showed that these nano-molecules entrap the viral pathogens and also mediate their clearance from body fluids, preventing the spread of infections (113–115). The mechanism of viral entrapment by lung-derived EVs involves binding of SARS-CoV-2 viral spike protein (S protein) with the ACE-2 receptor available on these EVs, so that viral S protein does not participate in binding with the human ACE-2 receptor, but binds with EVs-ACE-2 receptors, thereby protecting the spread of SARS-CoV-2 infection and reducing the associated respiratory complications (116, 117).

### 7.1 Targeting mechanism of quercetin against SARS-CoV-2

SARS-CoV-2 is a highly diverse enveloped positive-sense single-stranded RNA virus that follows the entry into the human *via* angiotensin-converting enzyme 2 receptors (hACE2) present on the epithelial cells. The initial entry step for entry involve binding of its spike (S) protein to these receptors including human amino peptidase N (APN; HCoV-229E), angiotensin-converting enzyme 2 (ACE2; HCoV-NL63, SARS-CoV and SARS-CoV-2) and dipeptidyl peptidase 4 (DPP4; MERS-CoV) (118). The S proteins of the coronavirus are glycoproteins that exhibit S1 and S2 domains (119, 120). Moreover, the S1 domain comprehends the receptor-binding domain (RBD) that specifically recognizes host epithelial cell receptors. S2 domain exhibits heptad repeat sequences along with fusion peptides which assist fusion of viral and host cell membranes undergoing rearrangements mechanism (121). QCT is a widely known flavonoid and exhibits anti-COVID-19 activity and protective mechanism against SARS-CoV-2 through inhibition and triggering down regulation of hACE-2 along with discharge of pro-inflammatory chemokines.

Furthermore, QCT-3-b-D-glucoside also known to inhibit the expression of 3CLpro (also referred to as the main protease) and papain- like protease (PLpro) as quoted by authors of recently published study (122). The molecular docking study revealed that anti-SARS activity of QCT is demonstrated by the inhibition of SARS-CoV-2 protease through fabricating new hydrogen bonds with amino acid residues including His164, Glu166, Asp187, Gln192, and Thr190) of 6LU7 (123, 124). QCT known to exhibit >80.0% inhibition in an *in-vitro* experiment, with IC50 value of 73 μM, on the recombinant 3CLpro protein expressed in Pichia pastoris as reported by authors of previously published study (125). Another molecular docking observation revealed that QCT interacts with Asp38 of hACE-2 and inhibit the entry into the epithelial cells by preventing attachment of viral spike S1 protein. Some previously published studies showed the therapeutic role of EVs as anti-viral in many diseases (126–128).

## 8 Conclusions

Altogether, it is evident that QCT and EV supplementation significantly help to combat inflammatory responses, including SARS-CoV-2 infection. Here we show that QCT can be prospectively used in the form of nanoparticles after loading into the EVs to address the therapeutic potential in the COVID-19 pandemic. QCT nanoparticles can be loaded into the EVs using a suitable method of engineering after optimization of the efficiency and yield among the several physical and chemical methods available for engineering EVs. The quercetin nanoparticles loaded EVs act in two ways: (a) QCT will aid in combating the associated complications with SARS-CoV-2, such as inflammation, reactive oxygen species, and the regulation of genes involved in the release of cytokines and pro-inflammatory cytokines; and (b) the presence of ACE-2 receptors on the lungs derived EVs will show strong binding affinity with the S protein of the SARS-CoV-2 virus and thus As a result, developing QCT-loaded EVs as a therapeutic drug delivery approach is a better option to investigate for the treatment of viral and inflammatory diseases, such as SARS-CoV-2 infection. Several pre-clinical and clinical trials within this domain are needed to make this prospective therapy into a translational aspect within clinics. QCT is a GRAS molecule as per the USFDA guidelines and accordingly it may be exploited along with the EVs as antivirals and as drug constituent to control the symptoms of the COVID-19. In future research is needed to further explore the different types of cell sources derived EVs containing the QCT nanoparticles for effective treatment of comorbidities associated with the COVID-19. Further translational therapeutics can be developing in the form of oral and also inhaler medicine using this therapeutics.

## Author contributions

Conceptualization, AR, G-BJ, RG, and SK. Methodology, AR and G-BJ. Software, AR. Validation, G-BJ, RG, and SK. Formal analysis, G-BJ, RG, and SK. Investigation, AR. Resources, SK. Data curation, AR. Writing—original draft preparation, AR. Writing—review and editing, RG, SA, G-BJ. Visualization, RG and SK. Supervision: RG, G-BJ, and SK. Project administration, SK. All authors contributed to the article and approved the submitted version.
